# Identification and reconstruction of novel antibiotic resistance genes from metagenomes

**DOI:** 10.1186/s40168-019-0670-1

**Published:** 2019-04-01

**Authors:** Fanny Berglund, Tobias Österlund, Fredrik Boulund, Nachiket P. Marathe, D. G. Joakim Larsson, Erik Kristiansson

**Affiliations:** 1 Department of Mathematical Sciences, Chalmers University of Technology, Gothenburg, Sweden; 2 Centre for Antibiotic Resistance Research (CARe), University of Gothenburg, Gothenburg, Sweden; 3 Center for Translational Microbiome Research (CTMR), Department of Microbiology, Tumor and Cell Biology, Karolinska Institutet, Stockholm, Sweden; 4 Department of Infectious Diseases, Institute of Biomedicine, the Sahlgrenska Academy, University of Gothenburg, Gothenburg, Sweden; 5 Institute of Marine Research (IMR), Bergen, Norway

**Keywords:** Antibiotic resistance, Resistome, Environmental sequencing, Gene assembly, Microbiome, Beta-lactamases

## Abstract

**Background:**

Environmental and commensal bacteria maintain a diverse and largely unknown collection of antibiotic resistance genes (ARGs) that, over time, may be mobilized and transferred to pathogens. Metagenomics enables cultivation-independent characterization of bacterial communities but the resulting data is noisy and highly fragmented, severely hampering the identification of previously undescribed ARGs. We have therefore developed fARGene, a method for identification and reconstruction of ARGs directly from shotgun metagenomic data.

**Results:**

fARGene uses optimized gene models and can therefore with high accuracy identify previously uncharacterized resistance genes, even if their sequence similarity to known ARGs is low. By performing the analysis directly on the metagenomic fragments, fARGene also circumvents the need for a high-quality assembly. To demonstrate the applicability of fARGene, we reconstructed β-lactamases from five billion metagenomic reads, resulting in 221 ARGs, of which 58 were previously not reported. Based on 38 ARGs reconstructed by fARGene, experimental verification showed that 81% provided a resistance phenotype in *Escherichia coli*. Compared to other methods for detecting ARGs in metagenomic data, fARGene has superior sensitivity and the ability to reconstruct previously unknown genes directly from the sequence reads.

**Conclusions:**

We conclude that fARGene provides an efficient and reliable way to explore the unknown resistome in bacterial communities. The method is applicable to any type of ARGs and is freely available via GitHub under the MIT license.

**Electronic supplementary material:**

The online version of this article (10.1186/s40168-019-0670-1) contains supplementary material, which is available to authorized users.

## Background

Infections caused by antibiotic resistant bacteria are increasing globally, providing a major threat to public health [1]. Antibiotic resistance can be an intrinsic characteristic of a bacterial species, but in its clinical meaning, it is a trait acquired via mutations in pre-existing chromosomal DNA or, more commonly, via horizontal transfer of genes [2]. Environmental communities harbor a large diversity of antibiotic resistance genes (ARGs) which can, if mobilized, spread to pathogens either directly or via commensal bacteria in human and animals [3–5]. Indeed, many of the clinically relevant ARGs are believed to originate from environmental bacteria, together constituting a large and almost unexplored resistance reservoir [6]. Furthermore, it has been shown that strong selection pressures can enrich the abundance and diversity of ARGs [7], a phenomenon that has been especially prominent in environments exposed to high concentrations of antibiotics [8–10]. It is therefore vital to investigate the resistome in environmental and commensal bacterial communities, including the large number of uncharacterized ARGs. This will increase the understanding of the processes behind the evolution and mobilization of ARGs into human pathogens and facilitate early surveillance and confinement actions before they reach clinical settings.

Shotgun metagenomics enables analyses of bacterial communities through sequencing of random fragments of the collected genomes [11, 12]. Less than 1% of environmental bacterial species are considered cultivable using standard methods. Metagenomics therefore provides a complementary view of a community, including its ARGs, in comparison with cultivation-based approaches [13]. With the increasing capacity of massively parallel sequencing, shotgun metagenomics has rapidly become cheaper to perform, and data have accordingly become more accessible [14]. However, metagenomic data is highly fragmented and is characterized by a wide range of artifacts and noise [15]. This makes the assembly of larger genomic regions a difficult undertaking, especially for less abundant bacterial species and strains. This is especially true for ARGs, which are often present in regions with repetitive elements and multiple contexts in, e.g., integrons, transposons, and plasmids. ARGs are therefore notoriously hard to reconstruct from metagenomic data [8, 16, 17]. Therefore, a large diversity of previously uncharacterized ARGs in metagenomic datasets are likely to be overlooked by existing analysis pipelines.

Identification of antibiotic resistance genes in bacterial DNA sequences is often done through alignment-based homology searches against an ARG reference database [18–20]. Several reference databases have been designed for this purpose, including ARDB [21], SARG [22], CARD [23], and ResFinder [20]. Even though these and other reference databases contain thousands of characterized ARGs, they only reflect a small proportion of the total resistome [6]. Moreover, the vast majority of the annotation pipelines are developed to find known ARGs and are often not optimized, or even able, to accurately identify functional resistance genes with low similarity to known ARGs. This is especially true for metagenomic data which, due to the short fragment length, is especially hard to annotate [24–27]. Several methods have been developed for this purpose, for example, ARGs-OAP [22], GROOT [28], AmrPlusPlus [29], MEGAN [30], and ARIBA [31]. However, these methods only consider reads that can be stringently matched to a database with known ARGs. The accuracy of existing methods for finding metagenomic reads from completely novel genes is therefore unknown and, potentially, low. Other approaches for detecting ARGs are Resfams [32], which uses hidden Markov models for increased sensitivity, and PCM [33], which applies machine learning to incorporate information about the protein structure. These methods are however designed to work only with longer fully assembled gene sequences. Recently, deepARG was published as the first method designed to find novel ARGs directly from shotgun metagenomic data. deepARG uses a classifier based on artificial neural networks which makes it able to operate directly on short sequence fragments [34]. However, deepARG lacks the functionality to assemble the identified fragments into full-length ARG sequences. Thus, taken together, there are no methods for exploration of the resistome that can identify metagenomic reads from novel ARGs and then accurately reconstruct their full sequences.

Here we present fARGene, a novel method for identification and reconstruction of ARGs directly from metagenomic data. fARGene uses probabilistic gene models optimized to accurately identify previously uncharacterized resistance genes, even if they have a low sequence similarity to known ARGs. The method operates by first identifying fragments potentially originating from ARGs which then are reconstructed into full-length genes. The method is computationally efficient and does not require a complete assembly of the entire metagenome, which makes it applicable to very large sequence datasets. To demonstrate the method, ARG models representing all four β-lactamase Ambler classes (A, B, C, and D) were created and applied to five metagenomic datasets comprising more than five billion DNA reads. This resulted in 221 reconstructed ARGs, of which 58 were previously not reported (< 70% sequence similarity to any gene in NCBI Genbank). Furthermore, based on 38 experimentally validated novel ARGs previously reconstructed by fARGene and tested in earlier studies, > 80% were functional when expressed in *Escherichia coli*. Finally, we show that fARGene has superior performance compared to deepARG and five other methods with a significantly higher sensitivity for detecting novel β-lactamases. fARGene is applicable to any class of ARGs and is freely available via GitHub (https://github.com/fannyhb/fargene) under the MIT license, together with documentation and a tutorial.

## Results

**Fig. 1:**
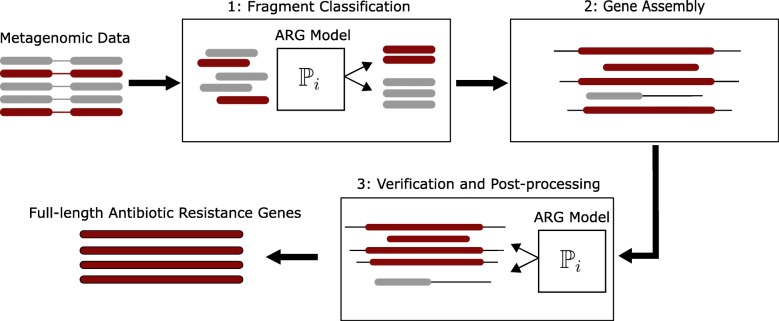
A schematic overview of fARGene. fARGene takes metagenomic paired-end data as input which then are subjected to an ARG model which classify the reads as coming from a resistance gene or not (panel 1). The paired-end sequences of the positively classified reads are extracted, quality controlled and then assembled into full-length genes (panel 2). The produced gene sequences are once again classified by the ARG model (panel 3). The output consists of nucleotide and amino acid sequences of the reconstructed ARGs. The method can also be applied directly to whole genomes and metagenomic contigs and then the classification, extraction, and assembly of reads are not performed

The method can be summarized into three main steps (Fig. 1). fARGene starts with fragmented metagenomic data as input, which is translated into amino acid sequences in all six reading frames. Next, an ARG model based on Hidden Markov models (HMM), describing the conserved sequence patterns of the class of resistance genes of interest, is used to score and classify each metagenomic read (panel 1, Fig. 1). The ARG model has been specifically optimized for finding novel ARGs in short read sequence data (see below). Reads that are identified as potentially originating from a class of ARGs are retrieved together with their respective read-pair, quality assessed, and then reconstructed into full-length sequences using paired-end assembly (panel 2, Fig. 1). The reconstructed sequences are quality assessed by classification again, and this time using an ARG model optimized for full-length genes (panel 3, Fig. 1). Open reading frames are finally predicted and nucleotide and amino acid gene sequences extracted. The method can also be applied directly to whole genomes and assembled metagenomic contigs, but in this case, the first step uses a gene model optimized for full-length genes and the assembly step is not performed.

The method also contains functionality to create ARG models and optimize their significance thresholds (Additional file 1: Figure S1). The optimization of the model aims to achieve high sensitivity to detect metagenomic fragments from ARGs, while at the same time maintaining as high specificity as possible. The specificity is here defined as the ability to *not* classify fragments from evolutionarily closely related genes without a resistance phenotype as ARG fragments. The ARG models are created based on a reference set containing experimentally validated resistance genes from the class of interest. The sensitivity is estimated using leave-one-out cross-validation where genes are consecutively excluded from the model, randomly fragmented, and then classified. The specificity is estimated based on fragments from a negative set of amino acid sequences from evolutionary closely related genes that does not induce a resistance phenotype. Both the sensitivity and specificity are then calculated from the proportion of correctly classified fragments. The optimal ARG model threshold score is then finally set based on the trade-off between sensitivity and specificity. Please refer to the “Methods” section and Additional file 2: Figure S2 for full details about the implementation of fARGene.

**Fig. 2:**
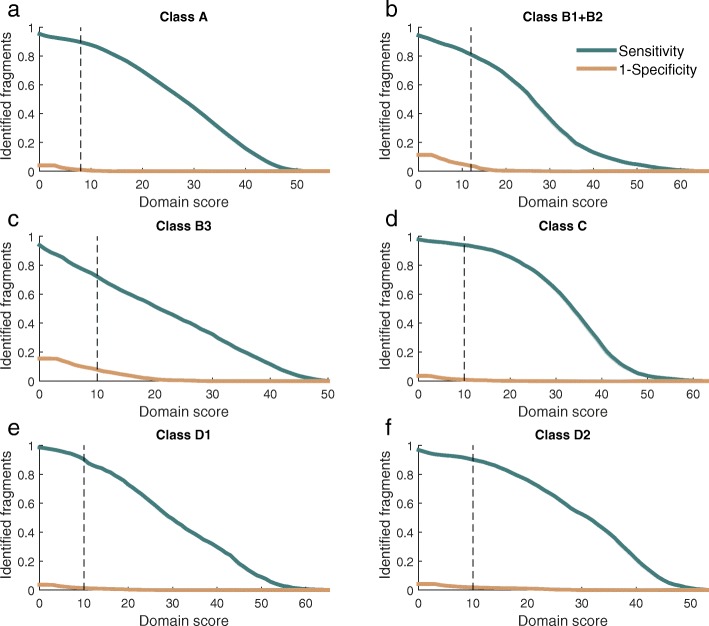
**a**–**f** Results from optimization of six ARG models for the four classes of β-lactamases. Each figure shows the performance of correctly classifying fragments as ARGs. The green curve shows the sensitivity, i.e. the fraction of correctly classified fragments from true resistance genes, while the orange curve shows 1-specificity, i.e. the fraction of incorrectly fragment sequences from genes without a resistance phenotype. A model with good performance should have a high sensitivity while 1-specificity should be low. The dashed line corresponds to the model threshold selected to have a high sensitivity and an acceptable specificity

As a case study, we used fARGene to reconstruct known and novel resistance genes in metagenomic data generated by short read DNA sequencing technology. For this aim, we created and optimized six models capturing the vast majority of genes within the four β-lactamase classes A, B, C, and D (Additional file 3: Table S1). β-Lactamases constitutes a diverse set of resistance genes that have a high clinical relevance, which makes them suitable to evaluate the performance of fARGene under different scenarios. The class B β-lactamases where separated into two models to correctly describe their parallel evolution [35]. Also, class D β-lactamases were separated into two models to fully capture the large diversity among these genes (Additional file 4: Figure S3). All models demonstrated perfect sensitivity and specificity for full-length genes (Table 1). For classification of 100 nucleotide long reads, which clearly is a more challenging task, there was a trade-off between achieving high sensitivity while still maintaining high specificity (Fig. 2 and Additional file 5: Figure S4). The threshold scores for all models except for B3 β-lactamases were set so that the sensitivity was high (0.94 to 0.81) while maintaining a specificity above 0.95. For the B3 β-lactamases, which have few known reference genes, a sensitivity ~ 0.70 was necessary to achieve a specificity above 0.90 (Fig. 2c). The specificity of the model was further validated by analyzing (1) the human genome, (2) sequence reads from the human genome, and (3) simulated metagenomic reads from 100 bacterial chromosomes where the beta-lactamases had been removed, for which fARGene did not identify a single false positive (see the “Methods” section for full details).

**Table 1:** Model performance

		Sensitivity		Specificity	
Model	Reference genes	Full-length	Reads (100 nt)	Full-length	Reads(100 nt)
Class A	71	1.000	0.897	1.000	0.990
Subclass B1 + B2	35	1.000	0.811	1.000	0.962
Subclass B3	11	1.000	0.722	1.000	0.921
Class C	22	1.000	0.939	1.000	0.991
Class D1	9	1.000	0.904	1.000	0.986
Class D2	20	1.000	0.901	1.000	0.981

**Fig. 3:**
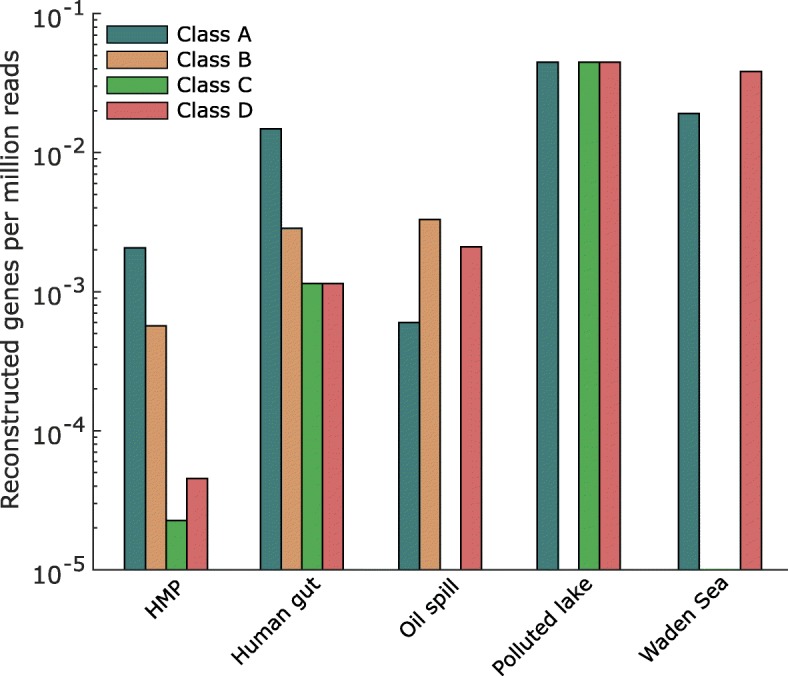
Number of reconstructed ARGs per millions reads for the four β-lactamase classes

Next, fARGene, using the optimized models, was applied to five metagenomic datasets comprising approximately five billion reads (Table 2). In total, 221 (212 unique) full-length ARGs were reconstructed, whereof 163 (74%) were known and 58 (26%) were novel (< 70% amino acid sequence identity to any previously reported gene in NCBI Genbank) (Table 3). The highest relative abundance of full-length genes for class A, C, and D β-lactamases was found in the metagenome from an antibiotic-polluted Indian lake [8] (Fig. 3). For class B β-lactamases, the highest relative abundance was instead found in oil-contaminated deep-sea metagenomes [36]. Both datasets from the human microbiome demonstrated high abundance of genes from class A and B β-lactamases while the levels were lower for class C and D. The highest relative number of novel genes were discovered in the oil-contaminated deep-sea metagenome (17 of 20 genes were novel). Furthermore, class D β-lactamases had the highest relative number of novel genes among the four classes (8 of 18, 44%). A list of all reconstructed genes and their sequences is available as Additional file 6: Table S2.

**Table 2:** Datasets used in this study

Dataset	Size (nt)	# reads	Avg. read length	Reference
HMP*	4.69 × 10^12^	4.41 × 10^10^	96	[12]
Human gut	2.80 × 10^11^	3.50 × 10^9^	75	[62]
Oil spill	3.36 × 10^11^	3.33 × 10^9^	101	[36]
Polluted lake	6.76 × 10^9^	6.69 × 10^7^	101	[8]
Wadden sea	8.42 × 10^9^	5.23 × 10^7^	161	[63]

*Human Microbiome Project

**Table 3:** Results from reconstruction of ARGs

	Class A		Class B		Class C		Class D	
	Reconstructed genes		Reconstructed genes		Reconstructed genes		Reconstructed genes	
	Total	New^†^	Total	New^†^	Total	New^†^	Total	New^†^
HMP^*^	91	23	25	8	1	0	2	0
Human gut	52	6	10	3	4	0	4	1
Oil spill	2	1	11	9	0	0	7	7
Polluted lake	3	0	0	0	3	0	3	0
Wadden Sea	1	0	0	0	0	0	2	0
Total	149	30	46	20	8	0	18	8

*Human Microbiome Project

^†^< 70% sequence similarity against any sequence in NCBI GenBank

Next, we applied fARGene to both metagenomic reads and assembled contigs to compare the results. Five samples were randomly selected from the Human Microbiome Project dataset and then analyzed by fARGene. The five samples were also assembled and the resulting contigs were then analyzed with fARGene (see the “Methods” section). For class A β-lactamases, which were the most abundant genes in these samples, 60 genes were predicted from the assembled contigs while 59 genes were predicted when fARGene was applied directly to the reads. Of these were 55 genes identical while five and four of the genes were uniquely identified from the contigs and the reads, respectively. The corresponding number for the class B β-lactamases were 11 and 10, respectively, where nine identical pairs were formed. We noted, however, that five of the genes differed in length and were ~ 10 amino acids shorter when reconstructed directly from the reads. No genes were reconstructed for class C and D β-lactamase using any of the approaches.

fARgene has previously been applied to reconstruct ARGs in three separate studies [37–39]. In conjunction with the respective study, a total of 38 predicted novel genes were phenotypically tested by synthesis of the gene sequence followed by cloning and expressing the gene in an *Escherichia coli* host. Of the 38 tested genes, 31 (82%) were functional in this host. This includes 18 of 21 (86%) novel metallo-β-lactamase from subclass B1 [37], 6 of 9 (67%) novel *qnr* genes [38], and 7 of 8 (88%) novel metallo-β-lactamases from subclasses B2 and B3 [39]. It should be noted that all genes were tested using their exact assembled sequence and several of the genes that failed to induce a resistance phenotype contained codons that are rare in *E. coli*, suggesting that they may still be functional in other hosts.

**Fig. 4:**
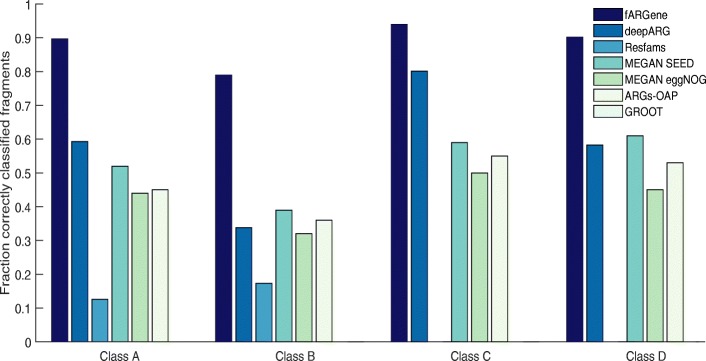
The ability to correctly classify metagenomic fragments for fARGene and five competing methods. The performance of fARGene was consistently higher than all compared methods (in average, 87% compared to 55%, 7.5%, 52%, 42%, 46%, and 0%, for deepARG, Resfams, MEGAN SEED, MEGAN eggNOG, ARGs-OAP, and GROOT, respectively)

**Fig. 5:**
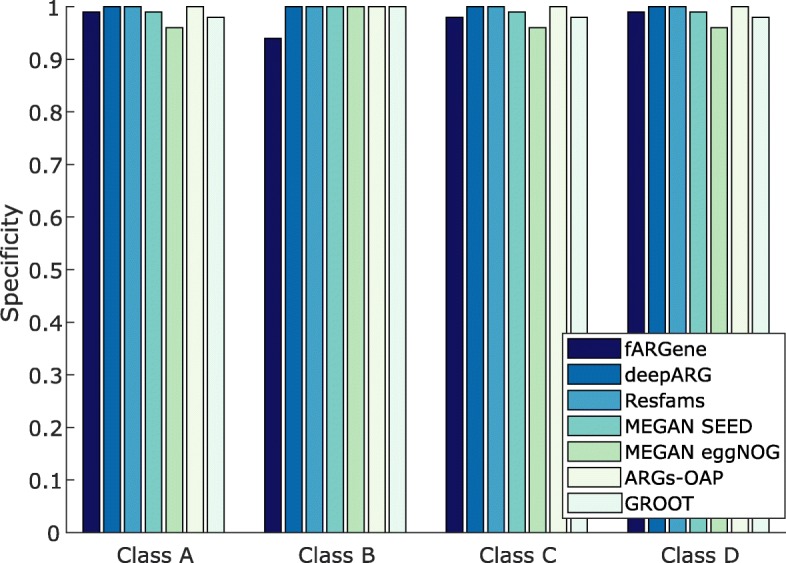
The estimated specificity for fARGene and five competing methods. The specificity was estimated from simulated metagenomic fragments of genes closely related to β-lactamases

Next, we compared fARGene’s ability to identify novel ARGs in metagenomic data with five competing methods: deepARG [34], Resfams [32], ARGs-OAP [22], GROOT [28], and MEGAN [30]. In contrast to fARGene, these methods do not have any functionality to reconstruct previously uncharacterized genes from metagenomic data and therefore we only evaluated the ability to correctly classify fragments from novel ARGs. For fARGene, deepARG, ARGs-OAP, and GROOT, known ARGs were removed (using clusters of 70% sequence similarity) from the databases/models of the methods, and we then measured the ability to correctly classify 100 nucleotide-long fragments of the excluded genes. This procedure was repeated for a total of 168 genes from the four classes of β-lactamases. For Resfams and MEGAN, no genes could be excluded since there was no functionality to efficiently rebuild the models/database (see the “Methods” section). The results showed that fARGene was able to correctly identify substantially more fragments of all β-lactamase classes, thus suggesting that it has superior sensitivity (Fig. 4). On average, fARGene had a sensitivity of 0.87 compared to 0.55 for deepARG and 0.52 for MEGAN (SEED database), which were the second and third best methods. The difference between fARGene and the other methods was especially large for ARGs belonging to class B β-lactamases, where fARGene and the second-best method for this class (MEGAN SEED database) had a sensitivity of 0.79 and 0.39, respectively. Taking the New Delhi metallo-β-lactamase (NDM) gene as an example [40], fARGene was able to correctly identify 78% of the fragments while the highest number among all the competing methods was only 4.9% (deepARG). The specificity was estimated for all methods by analyzing 100 bases long-nucleotide fragments of the close homologs in the negative dataset. Here, three of the methods, deepARG, ARGs-OAP, and Resfams, provided a specificity of 1 for all gene classes (Fig. 5). The specificity of GROOT, MEGAN eggNOG database, MEGAN SEED database, and fARGene was however slightly lower with average values of 0.98, 0.97, 0.99, and 0.98, respectively. In addition to the evaluation of short read classification for the abovementioned methods, we also evaluated the ability of ARIBA [31] to correctly reconstruct ARGs from metagenomic reads. Here, one ARG and sequences with more than 70% amino acid sequence similarity to that ARG were excluded from the database of ARIBA and the model of fARGene. Metagenomic reads were then simulated from the excluded ARG which were analyzed by ARIBA and fARGene. The results showed that fARGene correctly reconstructed all the excluded ARGs (168 of 168) while ARIBA was unable to reconstruct a single gene (0 of 168).

## Discussion

In this paper, we describe fARGene, a method developed to identify and reconstruct antibiotic resistance genes directly from metagenomic data. The method uses gene models which incorporates the evolutionarily conserved patterns of the ARG class of interest and optimizes their sensitivity and specificity using cross-validation. This ensures high performance, even for relatively short metagenomic fragments. The high accuracy is further supported by experimental validation where > 80% of the novel ARGs predicted by fARGene has shown to induce a resistance phenotype in *Escherichia coli*. In contrast to other methods, fARGene offers reconstruction of completely novel genes directly from sequence reads. fARGene can be applied to metagenomic sequence data generated by next-generation sequencing as well as longer sequences, such as bacterial genomes, plasmids, and assembled contigs. The complete method, including the functionality to design and optimize novel ARG models, is open source and freely available at GitHub under the MIT license.

The method was demonstrated by reconstructing ARGs from class A, B, C, and D β-lactamases from a comprehensive dataset consisting of metagenomes from both the environment and the human microbiome. More than 200 resistance genes were identified, whereof 74% were known while 26% were novel and not available in public sequence repositories. Novel ARGs were abundant in both the human microbiome and the environmental metagenomes and were particularly high in the oil-contaminated metagenome where 85% of the reconstructed genes were previously not reported. Thus, these results further support the hypothesis of a diverse and, to a large extent, uncharacterized resistome. Further exploration of ARGs, especially within uncultivable bacteria, is therefore warranted and will likely be crucial to understand how ARGs evolve, mobilize, and transfer into pathogens. Increased knowledge of ARGs is also important for more detailed phylogenetic studies, which is necessary to further elucidate their evolutionary history and origins [37, 41]. fARGene was developed to address these knowledge gaps and can, as demonstrated here, accurately reconstruct both known and novel ARGs from metagenomic fragments generated by sequencing of bacterial communities. fARGene thus provides the means to more thoroughly analyze the resistome, including its many unexplored parts that today are overlooked by existing methodologies. It should, however, be pointed out that fARGene only can discover genes that share some degree of homology with previously characterized ARGs. Genes that provide resistance through completely new resistance mechanisms and therefore have no sequence similarity to known ARGS will be overlooked. For this purpose, fARGene needs to be complemented with other molecular techniques such as functional metagenomics, where novel genes are screened based on the phenotype they provide, rather than their sequencing similarity [42, 43].

fARGene applies threshold scores that are optimized for each ARG model using leave-one-out cross-validation. This ensures that the method is able to correctly identify novel resistance genes (high sensitivity) but at the same time manage to correctly separate them from similar sequences without the desired phenotype (high specificity). The reference sequences used in the optimization should be well-characterized, sufficiently heterogeneous, and, preferably, have an experimentally verified resistance phenotype. For many forms of resistance genes, such sequences can be retrieved from comprehensive and well-curated databases such as CARD [23] and ResFinder [20]. It should, however, be emphasized that the gene models need to be created with some care in order to achieve as high sensitivity and specificity as possible. For example, the class of metallo-β-lactamases is known to have a high sequence diversity [44], and based on phylogenetic studies, the subclasses B1 and B2 are suggested to have developed the resistance phenotype independently of subclass B3 [35]. Thus, there are likely genes that does not result in a resistance phenotype (e.g., members of the much larger metallo-β-lactamase superfamily [45]) that are more evolutionary close to B1 and B2 than genes from B3 and vice versa. In fact, when all metallo-β-lactamases were combined into one single model, the sensitivity and specificity were reduced significantly (results not shown). Thus, classes of ARGs with parallel evolution and/or an overall high diversity may need to be separated into several models to ensure maximum performance.

Another important aspect in optimizing the ARG models and in achieving a correct specificity is to select an appropriate negative gene set. For example, the serine β-lactamases (A, C, and D) share a common ancestor with the DD-peptidases [46] which have a similar protein structure but lack the ability to hydrolyze β-lactams [47]. For the ARG models presented in this study, a comprehensive set of DD-peptidases were therefore selected as a negative set. Metallo-β-lactamases are, on the other hand, phylogenetically a part of the large metallo-β-lactamase (MBL) superfamily, whose members are related to various biological functions including mRNA processing and DNA repair [45]. Genes within the MBL superfamily that do not provide resistance to β-lactams were therefore chosen as a negative set. Thus, selection of a proper negative set requires knowledge about the evolutionary history of the resistance genes of interest. It should be underlined that estimating the specificity only from closely related genes that do not induce any resistance phenotype represents a “worse case” scenario. Indeed, most of the fragments encountered in metagenomes will originate from completely different genes that are much more evolutionary divergent. However, a critical and common error when predicting novel antibiotic resistance genes is to incorrectly assign a resistance phenotype to a closely related gene. We therefore argue that it is important to limit the false positives and thus calculate the specificity under as adverse conditions as possible. Finally, it should be emphasized that even though sensitivity and specificity of fARGene is high overall, the reconstructed genes should be considered as computational predictions. Experimental validation, preferably through expression of the predicted genes in a bacterial host, is necessary to ensure that the associated phenotype is induced.

fARGene was compared to six methods for classifying metagenomic reads, including deepARG which is a method that uses artificial neural networks for the identification of reads from novel ARGs. Based on the comparison, fARGene displayed 17% to 124% higher sensitivity to identify DNA fragments from novel genes than deepARG, which was the method with the second-best performance. The main reason for the high accuracy of fARGene is the use of more sensitive algorithms for sequence alignment. In particular, deepARG and MEGAN uses the alignment tool DIAMOND while ARGs-OAP uses USEARCH to match metagenomic fragments against a comprehensive reference database. Similar to the Basic Local Alignment and Search Tool (BLAST), DIAMOND and USEARCH are seed-based and have therefore a limited ability to detect more evolutionarily distant genes [48, 49]. Similarly, the methods GROOT and ARIBA take advantage of fast but highly stringent read mappers (a custom hierarchical local alignment using hashing-based indexing for GROOT and minimap [50] for ARIBA). All of these methods are therefore struggling to identify metagenomic reads from genes distantly related to the ones present in the database. In contrast, fARGene uses probabilistic ARG models in the form of profile Hidden Markov models (HMM), which specifically describe the evolutionary relationship between the ARGs of interest. Indeed, HMMs has been shown to have superior accuracy compared to many other alignment-based approaches [51]. Note, however, that the gene-specific optimization performed by fARGene is necessary to achieve a high performance. Resfams, which also are based on HMMs, had inferior performance to fARGene since their models are not adapted to analyze short DNA fragments from novel ARGs. It should, however, be pointed out that the high sensitivity comes at the expense of a slightly lowered specificity. This is addressed in fARGene through an extra validation step where reconstructed genes are again compared against the gene model. Since we did not observe a single misclassification when fARGene was applied to full-length genes, it is likely that any falsely reconstructed gene are removed at this stage. Furthermore, it should be noted that deepARG and many of the other general methods are, in contrast to fARGene, directly applicable to a wide range of ARGs without optimizing any significance thresholds. Thus, the superior performance provided by fARGene comes at the cost of the additional work needed to construct and optimize ARG models. In fARGene, this can, however, be done using the built-in model optimization functionality, which can be applied to any ARG class that may be of interest. It should finally be pointed out that the evaluation was done only for beta-lactamases and, even though this is a diverse group of genes, there is no guarantee that the performance, both in absolute terms and in relation to other methods, is as high for all forms of ARGs.

Identification and reconstruction of ARGs from metagenomic data are often computationally challenging. Short read DNA sequences generate an immense number of fragments which can require very large computational resources to assemble. In addition, ARGs are known to appear in many different genetic contexts, which makes them notoriously hard to assemble. Metagenomes are also typically under-sampled, and genes available only in a low abundance in the microbial community may therefore be present in the sequence data but, due to low coverage, missed by the assembly. fARGene circumvents the issues related to creating a quality assembly by operating directly on the metagenomic fragments. Instead, in the second step, an assembly is performed utilizing the paired-end structure of the data, but only on the reads that are classified as coming from ARGs and their read-pair. Thus, fragments from other forms of genes and DNA regions, which typically correspond to the vast majority of the metagenomic data, are excluded from the analysis. This results in a significantly reduced computational time. Furthermore, when comparing predictions of novel ARGs from fARGene applied directly to metagenomic reads to assembled contigs, the results were very similar. This shows that the more computationally efficient approach used by fARGenes does not lead to any considerable reductions in accuracy. Moreover, fARGene has been designed to fully utilize modern computer hardware where the input sequence data are split and processed simultaneously on multiple central processing units (CPUs). In fact, fARGene has a computational complexity that scales almost linearly to the number of reads (Additional file 7: Figure S5). fARGene can therefore process large metagenomic datasets (billions of reads) within a reasonable time on a standard computer.

## Conclusions

In conclusion, fARGene is a new method to identify novel ARGs directly from metagenomic data. In comparison to competing methods, fARGene offers both superior performance and the ability to reconstruct completely novel genes directly from sequence reads. fARGene thus facilitates the study of ARGs maintained by bacterial communities and provides the means to significantly expand the knowledge about the scope and diversity of the resistome. Further exploration of the resistome is absolutely key for an increased understanding of the evolutionary processes behind the development, mobilization, and transmission of resistance genes. This also facilitates early surveillance and the implementation of improved management strategies to prevent the spread of new forms of multiresistant bacteria.

## Methods

### Implementation of fARGene

The implementation of fARGene was done as follows. Metagenomic data was converted from FASTQ to FASTA using “seqtk” [52] with parameters “seq –a”. Then, genomic and metagenomic data was, if not already amino acid sequences, translated in six reading frames using EMBOSS transeq version 6.3.1 [53] with parameters “-frame=6 -table=11 sformat=pearson”. The translated sequences were then subjected to a profile HMM using HMMER3’s “hmmsearch” with parameters “-E 1000 --domE 1000 –domtblout”. The sequence ID, domain score, envelope start, and envelope end (“env_start,env_end”) were retrieved from the output of “hmmsearch” and used to classify sequencing depending on the alignment length and domain score. A whole genomic sequence was classified as positive if the domain score was higher than the optimized full-length threshold score. Fragmented sequences were classified using a length-dependent cutoff as shown below.

$$ \frac{\mathrm{domain}\ \mathrm{score}}{\mathrm{aligned}\ \mathrm{sequence}\ \mathrm{length}}>\frac{\mathrm{threshold}\ \mathrm{score}}{\mathrm{read}\ \mathrm{length}} $$

Based on the alignment coordinates (env_start, env_end), the predicted genes were extracted from the whole genomic input data using “seqtk subseq”. Finally, to obtain the open reading frames (ORFs) of the predicted genes, the predicted genes were extracted together with 200 bases extra on each side of the alignment (using the alignment coordinates given by “hmmsearch”). The extracted sequences were then subjected to Prodigal [54] with parameters “-f gff -p meta”. The predicted ORFs were once again analyzed with “hmmsearch” and then, if the predicted ORFs were longer than a model-dependent length cutoff, provided as both amino acid sequences and nucleotide sequences. For the metagenomic data, the reads that were classified as positives were retrieved with “seqtk subseq”, together with their read-pair in FASTQ. This was also done for reads where only one of the reads in the pair was classified as a positive. The extracted reads then proceeded to the quality control and adapter removal, performed with “Trim Galore!” version 0.4.1 [14] with parameter “--paired”. Then, the reads, including their paired ends, were assembled using “metaSPAdes” version 3.8.1 [55] and the assembled contigs were subjected to the profile HMM using “hmmsearch”. The output from the “hmmsearch” was parsed and the contigs were classified using the optimized full-length gene threshold score, and the genes classified as positives were retrieved with “seqtk subseq”, both as nucleotide and amino acid sequences. In addition to the retrieved sequences that aligned to the model, the assembled contigs were analyzed using NCBI ORFfinder, a tool better suited than Prodigal to be applied to the often relatively short contigs, with parameters “-outfmt 1 -ml 200 -s 1 -g 11”, and the predicted ORFs were then once again subjected to the profile HMM using “hmmsearch”. The ORFs that passed the full-length classification and were longer than a model-specific minimal ORF length were then provided as both nucleotide and amino acid sequences. The different gene models used in this study were processed separately.

The methods for model optimization using leave-one-out cross-validation were implemented as follows. First, one of the reference sequences from which the model was to be built of was excluded. The remaining reference sequences were aligned using ClustalW version 2 [56] with default parameters. The aligned sequences were then used to build a profile HMM with HMMER version 3.1b1 [57] using “hmmbuild” and “hmmpress” with default settings. To estimate the sensitivity of full-length genes, the excluded gene was analyzed with the profile HMM using HMMER3’s “hmmsearch”, parameters “--max -E 1000 --domE 1000 –domtblout”. This was repeated for each gene in the reference sequence dataset. The specificity was estimated by creating a profile HMM of all reference sequences using ClustalW version 2 and HMMER version 3.1b1, as described above. Then, a negative sequence dataset consisting of close homologs to the reference genes was chosen and analyzed with the model using “hmmsearch” with the above-described parameters. To obtain the optimal threshold score, the fraction of identified genes from both the reference and negative dataset was plotted as a function of the domain score from the analysis with “hmmsearch”. The threshold score was then chosen so that a complete separation of the reference and negative sequences was obtained.

To optimize the threshold score for fragmented metagenomic data, the estimation of sensitivity and specificity was performed once again, but each gene that was analyzed with the profile HMMs had been randomly fragmented into 10,000 reads of length 100 bases each. Here, a complete separation of the reference and negative reads was not the objective, but to keep the sensitivity sufficiently high without letting the specificity drop exceedingly.

Previously published work uses an early version of fARGene [37, 38]. The method presented in this paper has however a wide range of fundamental differences and optimizations. Briefly, the pipeline is now completely automated, and functionality for creation and optimization of customized gene models has been implemented. fARGene also makes full use of the paired-end structure of the data which significantly improves the gene reconstruction. Additional computational steps have been added to both the genomic and metagenomic version, including more accurate ORF prediction, targeted assembly of up and downstream regions around ORFs, more careful verification of reconstructed novel ARGs, and instant retrieval of full-length predicted genes as both nucleotide and amino acid FASTA sequences. Please refer to https://github.com/fannyhb/fargene for a complete summary of the functionality of fARGene.

### Development of the gene models and processing of data

To create the class A β-lactamase profile HMM, 104 reference sequences [58] were downloaded and clustered with a sequence identity cutoff of 70% using USEARCH version 8 [49], parameters “-cluster-fast -id 0.7”. The representative sequence for each cluster was then used to create the profile HMM. The negative sequences used to estimate the specificity were chosen from the close homologs DD-peptidases [47]. For the class B1 + B2 and B3 of the metallo-β-lactamases, 35 and 11 representative sequences, respectively, with a sequence similarity of < 70% to each other were chosen [37, 59] to build the profile HMMs, while the negative sequences were chosen from the closest known homologs, the metallo-β-lactamase superfamily [45]. The class C profile HMM was based on all reported plasmid-determined genes together with the chromosomal genes with a reported ampC-type β-lactamase activity [60]. These genes were clustered at a 70% sequence similarity cutoff as described for class A, and the resulting 22 representative sequences were used to create the model. For class D, all genes reported by Lahey Clinic (https://www.ncbi.nlm.nih.gov/pathogens/beta-lactamase-data-resources/) as OXA were clustered at 70% and the representative sequences for each cluster were used to create a phylogenetic tree. Based on the tree, the genes were divided into two groups here called class D1 and class D2, with 9 and 20 representative sequences, respectively, which were used to create two profile HMMs. In addition, the false positive rate was assessed by applying fARGene, one time for each model, to the complete human genome (hg19) and to raw Illumina reads retrieved from the 1000 genome project, study ERR129 (http://www.internationalgenome.org/). The false positive rate was furthermore assessed from 100 randomly chosen bacterial chromosomes that were shown to not contain β-lactamases by using the models optimized for full-length genes. The chromosomes were used to simulate metagenomic data with ART Illumina version 2.5 [61], parameters “-l 100 -f 300 -m 300 -qL 93 -s 0 -na -p” which was then analyzed with fARGene parameter “--meta”.

The data used in this study consisted of raw Illumina sequence reads and were human gut microbiome samples of 114 individuals [62], the HMP data which consisted of 764 samples from 16 different body sites [12], 14 samples from oil-exposed bacterial communities in marine sediments [36], one sample from a lake polluted by pharmaceuticals [8], and eight samples from a tidal flat in the German Wadden sea [63]. All datasets were processed with the above-described method, using the profile HMMs representing class A, B, C, and D β-lactamases with optimized threshold scores. The resulting predicted full-length genes were annotated using an in-house pipeline which runs BLAST+ version 2.6 [64] against NCBI non-redundant proteins and the resistance database Resqu (http://www.1928diagnostics.se/resdb), and genes that had less than 70% amino acid sequence identity to any previously reported gene were classified as novel.

To compare fARGene applied to sequence reads to fARGene applied to assembled contigs, five samples from HMP were randomly chosen (SRS011084, SRS011239, SRS011271, SRS013687, and SRS015578). These samples were then independently assembled using metaSPAdes. The resulting contigs were analyzed with fARGene using all β-lactamase models and the full-length threshold. Then, fARGene was applied to the metagenomic reads of the same five samples, one at a time using all models. The resulting predicted genes from the contigs and the sequence reads were then combined and clustered using an amino acid sequence similarity of 100% using USEARCH, parameters “-cluster-fast -id 1”.

### Comparison between fARGene and competing methods

To obtain a fair comparison between fARGene and the competing methods, the same representative genes used to build and optimize the models of fARGene were also used to estimate the performance of all methods. The comparison with Resfams was done by downloading the Resfams database from http://www.dantaslab.org/resfams/ and then using HMMER3’s “hmmsearch” with parameters “Resfams-full.hmm --cut_ga --domtblout” on 10,000 randomly created fragments from each of the genes used in the model creation of fARGene. The number of identified fragments was then calculated separately for each β-lactamase class. For the comparison against deepARG, one representative gene from any model was chosen and used as a seed to a DIAMOND BLASTp search against the deepARG database, parameters “-k 1000 --id 60 -e 1e-5 --sensitive”. Every sequence that had more than 70% sequence similarity to the representative gene were then removed from the deepARG database. The representative gene was then randomly fragmented into 10,000 reads of length 100 bases, which were analyzed using deepARG, parameters “--align --type prot --reads”. The procedure was then repeated for each representative gene of each model and the fraction of identified reads by deepARG was calculated for each β-lactamase class. To estimate the performance of MEGAN, all representative sequences of one β-lactamase class at a time were fragmented and compared to the NCBI NR database using DIAMONDBLASTp, parameters “-e 0.01”. The resulting DAA files from the DIAMOND BLASTp were meganized using MEGAN6 community edition, parameters “daa-meganizer -a2t prot_acc2tax-Nov2018X1.abin -a2eggnog acc2eggnog-Oct2016X.abin -a2seed acc2seed-May2015XX.abin”. The meganized DAA files were then loaded into MEGAN, and the number of correctly classified reads was calculated for each β-lactamase class using both SEED and eggNOG. Although MEGAN has the ability to perform a gene-centric protein-alignment assembly on the mapped reads, it includes no functionality for mapping against resistance databases such as CARD or Resfams and we therefore only evaluated the ability to correctly classify fragments.

The comparison against ARGs-OAP was done by first using one representative gene from any model as a seed to a BLAST+ BLASTp search against the SARG database (v. 2) with parameters “--max_target_seqs 1000” and sequences with more than 70% sequence similarity to the representative gene were removed from the SARG database. The representative gene was then fragmented into 10,000 simulated paired-end Illumina sequence reads of length 100 bases using ART Illumina version 2.5 [61], parameters “-l 100 -c 5000 -m 300 -qL 93 -s 0 -na -p”. The simulated metagenomic reads were then analyzed using the ARGs-OAP stage one pipeline “argoap_pipeline_stageone_version2”, and the number of retrieved reads were counted from the output file “extracted.fa”.

The comparison against GROOT was done against the Resfinder database [20] which was downloaded from https://bitbucket.org/genomicepidemiology/resfinder.git. One representative gene at a time was used as a seed to a BLAST+ BLASTn search against the Resfinder database, and sequences that had a sequence similarity of > 70% to the representative gene were removed while the excluded gene was fragmented into simulated paired-end reads with the same method as for ARGs-OAP. The modified Resfinder database was then clustered using VSEARCH version 2.10 parameters “--cluster size –id 0.9 --msaout MSA.tmp” and then ordered with the command “awk '!a[$0]++ {of="./cluster-" ++fc ".msa"; print $0 >> of ; close(of)}' RS= ORS="\n\n" MSA.tmp && rm MSA.tmp” following the GROOT manual. The reads were then analyzed with GROOT, parameters “align -f <reads_1.fq>,<reads_2.fq> | groot report -c 1”, and the number of identified reads was counted.

To compare the performance of fARGene with ARIBA, the CARD database was downloaded running ARIBA with parameters “ariba getref card”. Then the same procedure as for ARGs-OAP and GROOT was applied where one representative sequence at a time was used as a seed for a BLAST+ BLASTn search against the CARD database. Similar sequences were removed from the database and then the fragmented excluded sequence was analyzed with ARIBA, parameters “run <fastq1> <fastq2>”, and the number of reconstructed genes was counted. To get the corresponding number of reconstructed genes from fARGene, one representative sequence at a time was excluded from the HMM and then analyzed with fARGene, parameters “--hmm-model <modified_model.hmm> --meta” using the same threshold scores as were optimized for the complete models.

### Evaluation of the computational efficiency

Benchmarking how fARGene performance scaled with data size was done using subsets of the human gut data and with the class A β-lactamase model. The subsets consisted of 2, 4, 8, 16, 32, and 64 million reads, and the reads for each subset were taken from the forward and reverse sequences and split into one forward and one reverse subset file. For example, the two million reads subset consisted of two files with one million reads in each. Benchmarking was performed on a machine with 192 GiB of RAM using one CPU. Then, each subset was analyzed with the method, and the time was measured from input to the final assembled predicted genes.

### Functional verification of predicted genes

Of the in total 38 experimentally tested genes, 21 metallo-β-lactamase subclass B1 were predicted and tested by Berglund et al. [37], nine *qnr* genes were predicted and tested by Boulund et al. [38], two metallo-β-lactamase subclass B2 and seven metallo-β-lactamase subclass B3 were predicted and tested by Marathe et al. [39]. All genes were synthetized and then expressed in an *Escherichia Coli* host. For the novel metallo-β-lactamases verification, CarbaNP tests were performed while the *qnr* genes were assessed using ciprofloxacin minimum inhibitory concentration (MIC). Please refer to the respective study for detailed information.

## Additional files

Additional file 1: **Figure S1.** Flowchart of the model creation and optimization. (PDF 18 kb)

Additional file 2: **Figure S2.** A detailed view of the workflow of fARGene. (PDF 15 kb)

Additional file 3: **Table S1.** β-lactamases used to create all six models. (XLSX 18 kb)

Additional file 4: **Figure S3.** Phylogenetic tree of the clustered reference sequences of all OXA-type β-lactamases. (PDF 19 kb)

Additional file 5: **Figure S4.** ROC curves for the six β-lactamase models. (PDF 38 kb)

Additional file 6: **Table S2.** A list of all reconstructed and predicted genes together with their amino acid sequences. (XLSX 48 kb)

Additional file 7: **Figure S5.** Results from benchmarking of fARGene. (PDF 13 kb)
