# Supplementary material for: Identification and reconstruction of novel antibiotic resistance genes from metagenomes
Source: Microbiome. 2019 Apr 1;7:52. doi: 10.1186/s40168-019-0670-1 (PMC6444489; doi:10.1186/s40168-019-0670-1)
Supplement: Supplementary file 1 — Figure S1. Flowchart of the model creation and optimization. (PDF 18 kb) [file 40168_2019_670_MOESM1_ESM.pdf]

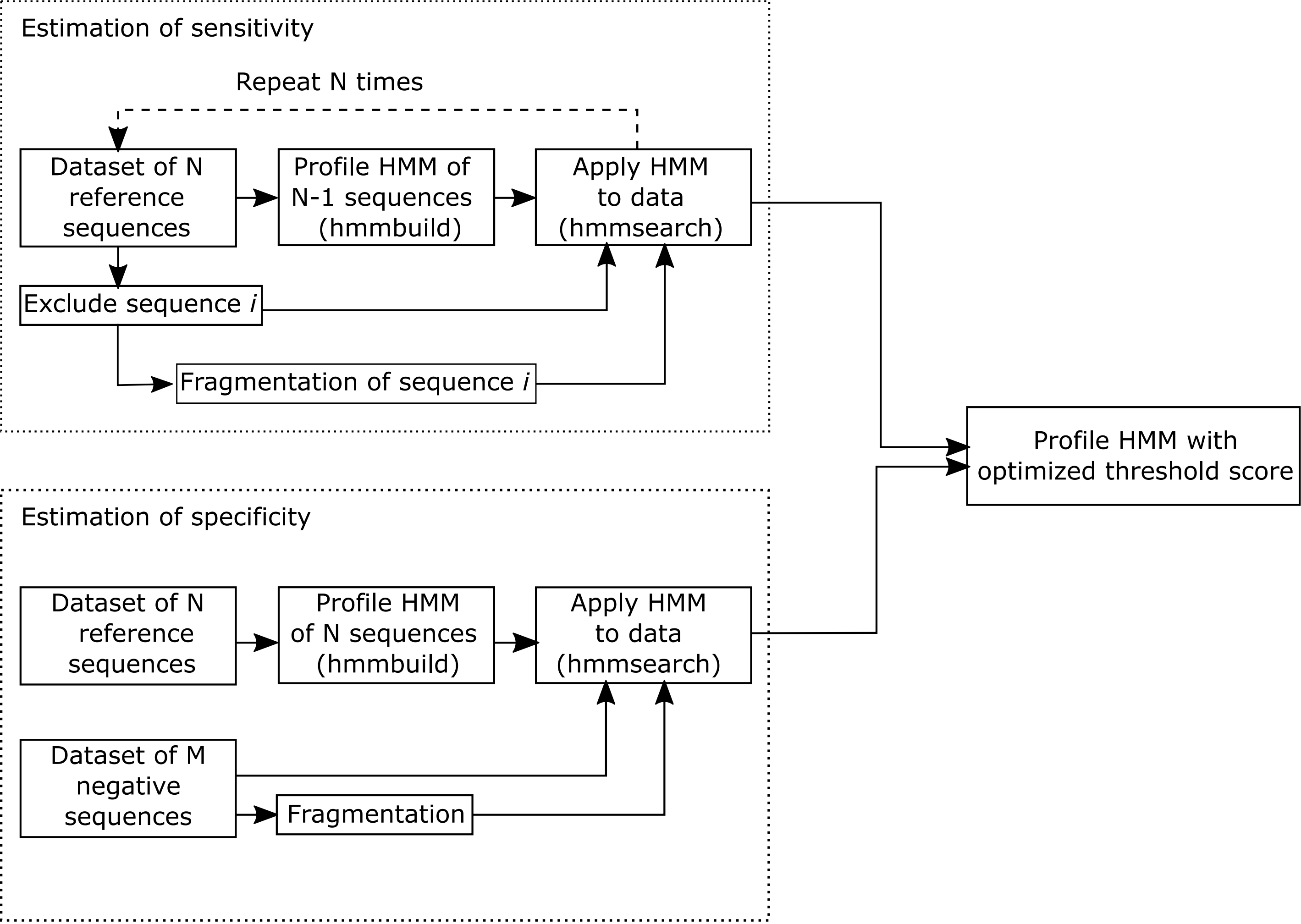
Estimation of sensitivity
Repeat N times
Dataset of N reference sequences
Profile HMM of N-1 sequences (hmmbuild)
Apply HMM to data (hmmsearch)
Exclude sequence *i*
Fragmentation of sequence *i*
Profile HMM with optimized threshold score
Estimation of specificity
Dataset of N reference sequences
Profile HMM of N sequences (hmmbuild)
Apply HMM to data (hmmsearch)
Dataset of M negative sequences
Fragmentation
