# Supplementary figures and images for: Identification and reconstruction of novel antibiotic resistance genes from metagenomes
Source: Microbiome. 2019 Apr 1;7:52. doi: 10.1186/s40168-019-0670-1 (PMC6444489; doi:10.1186/s40168-019-0670-1)

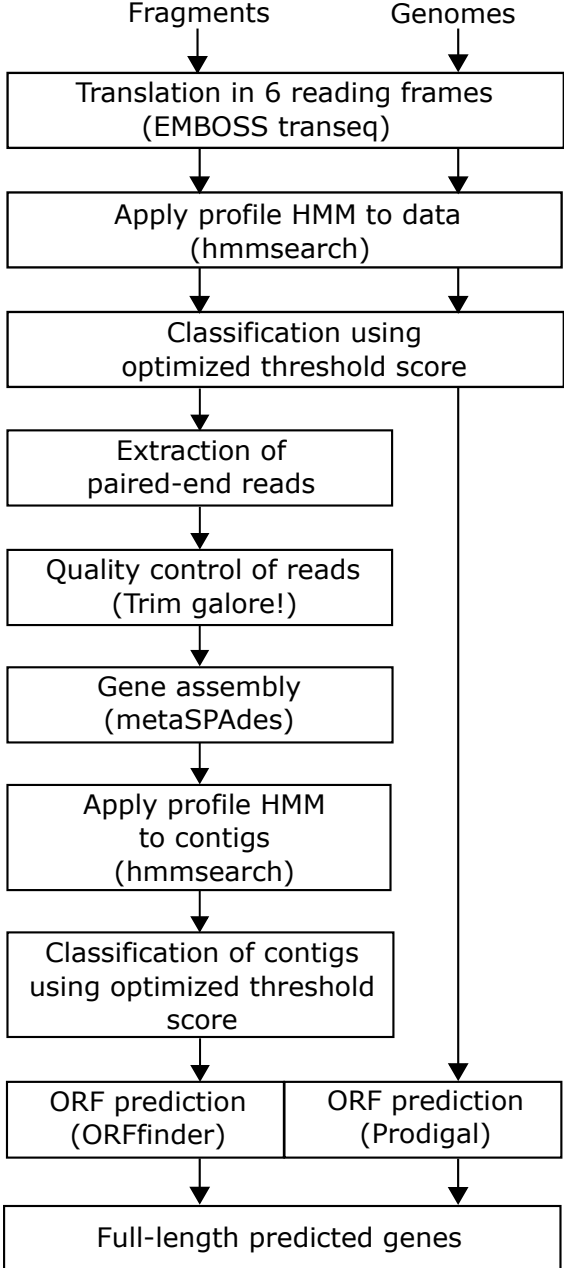

Supplement: Supplementary file 2 — Figure S2. A detailed view of the workflow of fARGene. (PDF 15 kb) [file 40168_2019_670_MOESM2_ESM.pdf]

Class D1

Class D2

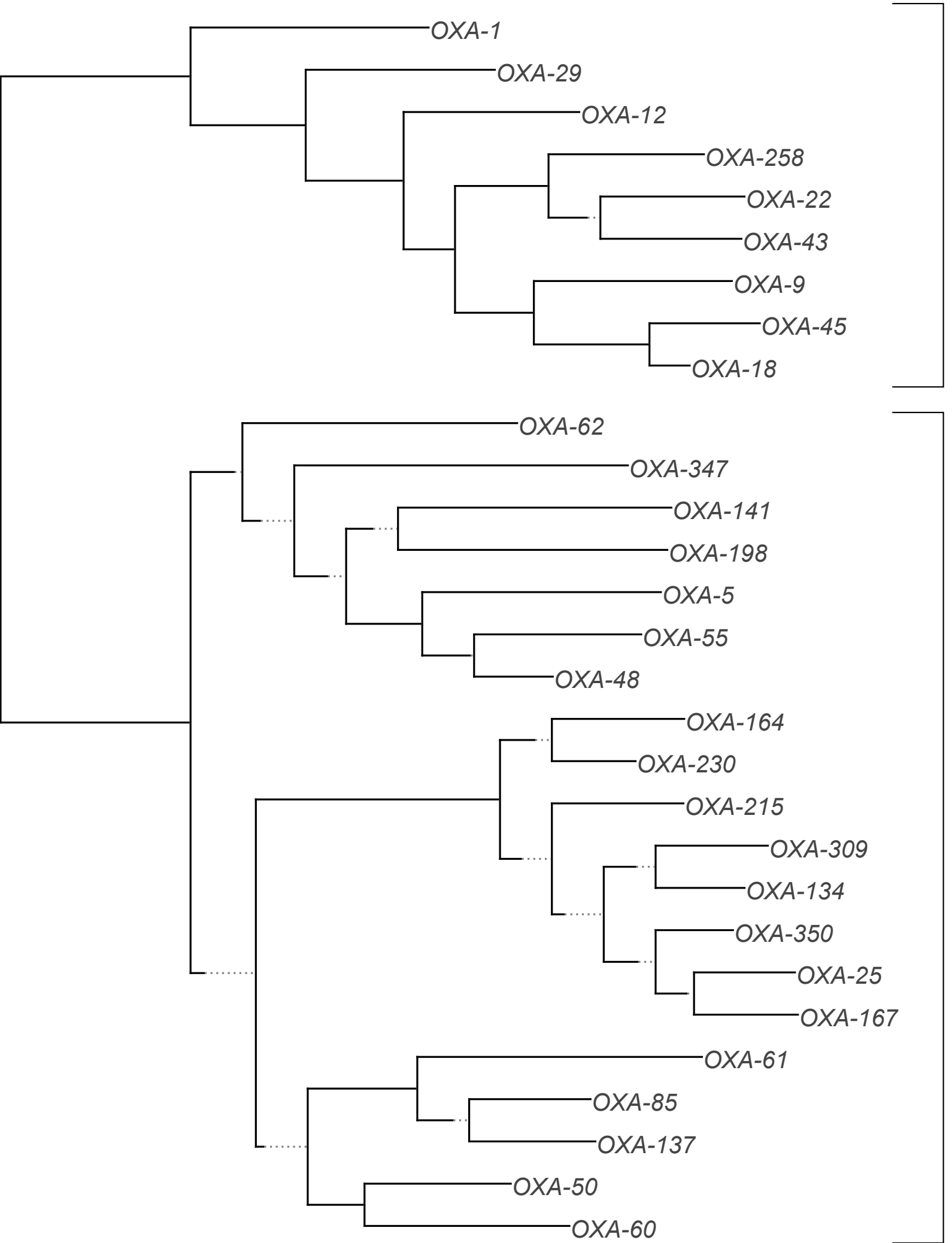

Supplement: Supplementary file 4 — Figure S3. Phylogenetic tree of the clustered reference sequences of all OXA-type β-lactamases. (PDF 19 kb) [file 40168_2019_670_MOESM4_ESM.pdf]

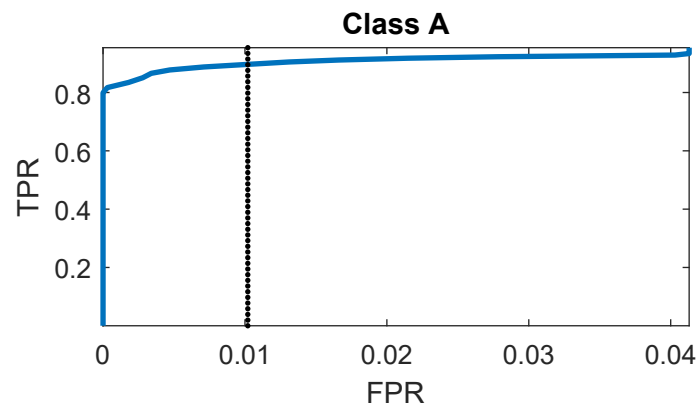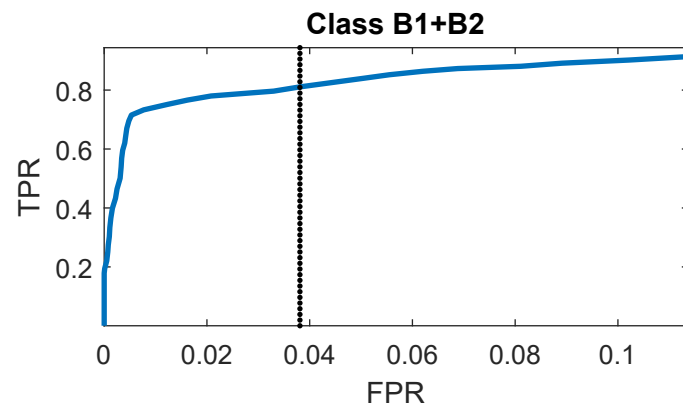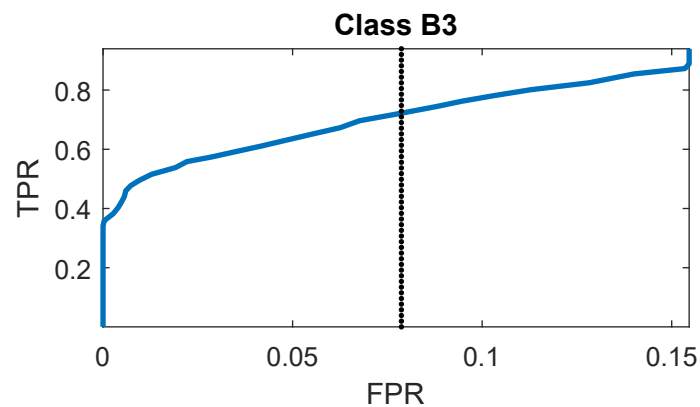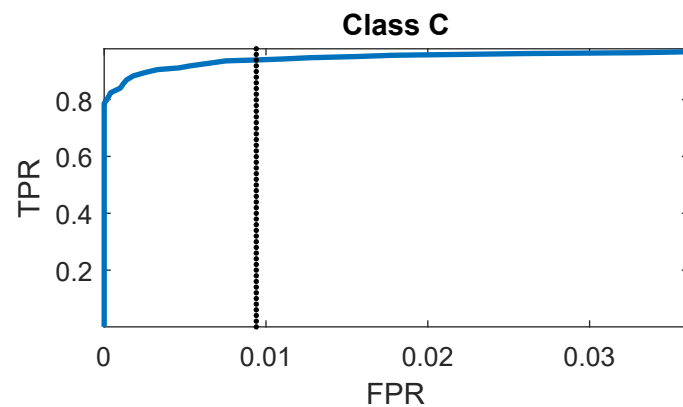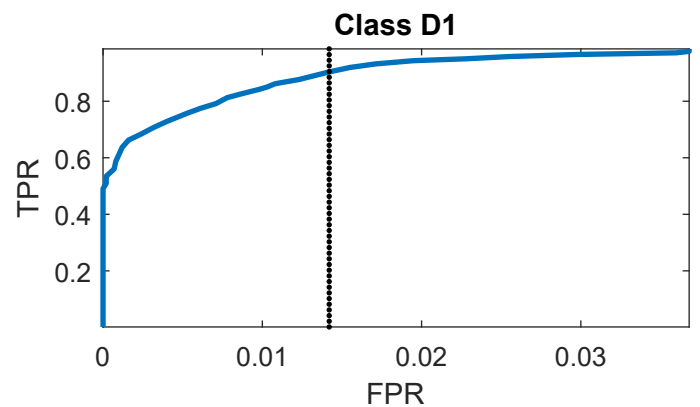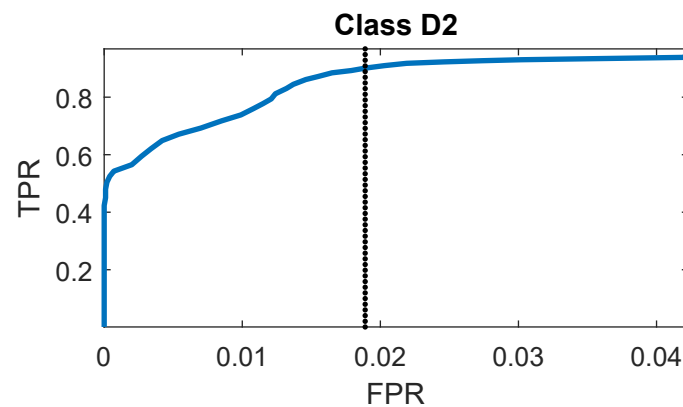

Supplement: Supplementary file 5 — Figure S4. ROC curves for the six β-lactamase models. (PDF 38 kb) [file 40168_2019_670_MOESM5_ESM.pdf]

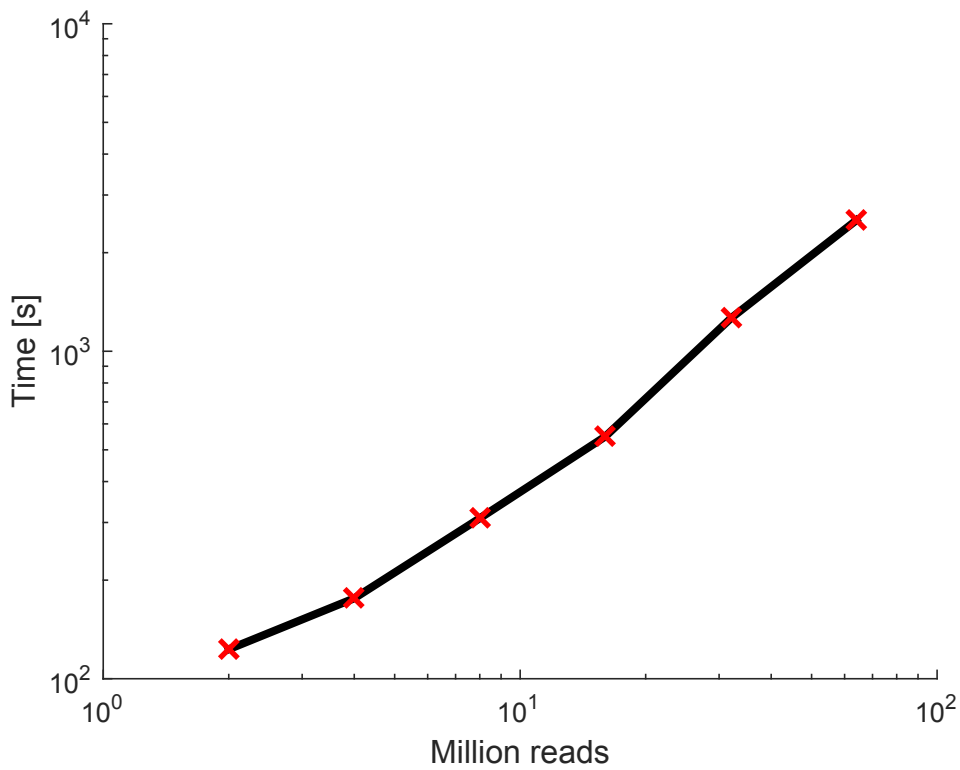

Supplement: Supplementary file 7 — Figure S5. Results from benchmarking of fARGene. (PDF 13 kb) [file 40168_2019_670_MOESM7_ESM.pdf]
